# Physiological mechanisms and organism response in youth sports activities-related musculoskeletal injuries

**DOI:** 10.3389/fphys.2026.1840665

**Published:** 2026-05-25

**Authors:** Lingchao Li, Kelei Guo, Guibing You, Jia Yang

**Affiliations:** 1 School of Physical Education and Health, Zhaoqing University, Zhaoqing, China; 2 Winter Olympic College, Harbin Sport University, Harbin, China

**Keywords:** adolescent athletes, exercise physiology, growth and maturation, injury prevention, musculoskeletal injury

## Abstract

Musculoskeletal injuries in young athletes arise within a biological context that is fundamentally distinct from that of adults, because rapid growth, physeal vulnerability, immature osteochondral structures, endocrine shifts, and asynchronous adaptation of bone, muscle, and tendon reshape both injury susceptibility and tissue recovery. This mini-review synthesizes current evidence showing that injury risk in youth sport is driven not by load alone but by the interaction between developmental biology and sporting exposure: growth-related weak points such as physes and traction apophyses predispose to unique injury patterns; low energy availability and hormonal disturbance can impair bone accrual and increase stress injury risk; and mismatched gains in muscle strength and tendon stiffness may amplify overuse pathology, particularly during periods of accelerated growth. These risks are further modified by skeletal maturity, sex, early sport specialization, psychosocial influences, and abrupt changes in training volume. Preventive strategies have therefore moved beyond generic advice towards neuromuscular training, education, and increasingly individualized load-management models, although important controversies persist regarding treatment thresholds for common injuries, the true benefits and harms of early specialization, and the extent to which screening and prevention should be sport-specific. Future progress in youth sports medicine will depend on a developmental, precision-oriented framework that integrates biomechanics, endocrinology, psychology, and implementation science to enable age-aware risk stratification, personalized prevention, and better long-term outcomes.

## 1 Introduction

Participation in youth sports activities holds irreplaceable value in promoting physical and mental health, cultivating team spirit, and establishing healthy lifestyle habits. However, the risk of musculoskeletal injuries associated with sports participation poses a significant challenge for public health and clinical medicine. Adolescents are not ‘miniature adults’; their unique growth and development physiology fundamentally determines differences in injury patterns, risk factors, and repair responses compared to adults (Parry et al., 2024; Putukian et al., 2026). Each year, numerous young athletes seek medical attention due to sports injuries, with popular sports such as football, basketball, and rugby bearing a particularly heavy injury burden (Mandorino et al., 2023; Lee et al., 2026). These injuries not only cause immediate pain and functional loss, forcing athletes to suspend training and competition, but may also have long-term impacts on growth and development, including skeletal deformities, accelerated joint degeneration, and significantly increased risk of future reinjury (Park et al., 2023).

Investigating the physiological mechanisms and organism responses underlying adolescent musculoskeletal injuries is fundamental to achieving effective prevention, precise diagnosis, and optimized management. The core of this research field lies in understanding how growing tissues, including bones, growth plates, tendons, and ligaments, respond to external mechanical loads, and how factors such as individual developmental stages, hormonal environments, and genetic backgrounds modulate the balance between adaptation and injury. For instance, during the adolescent growth spurt, rapid longitudinal bone growth combined with a transient imbalance in the muscle-tendon unit’s length-tension relationship is recognized as a key physiological basis for various overuse injuries, such as Osgood-Schlatter disease and Sever’s disease (Wedderkopp et al., 2026). In recent years, the cross-disciplinary integration of sports medicine, biomechanics, developmental biology, and exercise physiology has deepened academic understanding of adolescent sports injuries, shifting from purely phenomenological descriptions toward exploring molecular, cellular, and systemic-level mechanisms (Schlecht et al., 2026).

This mini-review synthesizes current research advances regarding physiological mechanisms underlying adolescent musculoskeletal injuries, focuses on key academic controversies, identifies existing knowledge gaps, and offers forward-looking recommendations for future research and clinical practice (**Table 1**).

**Table 1:** Key domains, mechanistic insights, and translational implications in youth sports-related musculoskeletal injuries.

Domain	Key points	Implications
Physiological basis	Weak physes and traction apophyses, rapid longitudinal growth, low energy availability, sex-hormone disturbance, and asynchronous muscle–tendon adaptation together create a distinctive injury-prone developmental milieu. Experimental work also suggests sex-related differences in ACL matrix damage and repair.	Assessment should be development-aware rather than based on age alone. Growth spurts, energy status, menstrual/endocrine health, and cumulative tendon loading should be monitored proactively.
Risk architecture	Risk reflects the interaction between early sport specialization, skeletal maturity, sex-related factors, sport-specific exposure, rapid load escalation, poor recovery, and previous injury. Clustering of these factors amplifies vulnerability.	Risk stratification should combine biological age, training volume, recovery pattern, and prior injury history rather than relying on chronological age alone.
Prevention and management	Neuromuscular training has the strongest evidence for improving movement quality, stability, and load tolerance. Education may improve symptom recognition and disclosure. Personalized load management and imaging-guided rehabilitation are emerging for selected high-risk groups.	Best practice is likely to combine universal prevention with subgroup-specific adaptation and embed programmes within routine training systems.
Academic controversies	Debate persists over surgical thresholds for selected injuries, the value of bracing in adolescent spondylolysis, the acceptable boundaries of early specialization, and whether prevention should use generic or context-specific protocols.	Management should remain individualized and sport-specific until stronger comparative effectiveness data are available.
Research gaps	Evidence is limited for psychosocial determinants of disclosure, biological mechanisms underlying sex differences, long-term adult sequelae of adolescent injury, and standardized diagnostic or classification systems.	Priorities include longitudinal cohorts, mechanistic translational studies, and consensus-based definitions, outcomes, and classification frameworks.
Future directions	The field is moving towards interdisciplinary, precision-oriented models integrating biomechanics, developmental biology, endocrinology, psychology, rehabilitation, wearables, and AI-assisted prediction, supported by implementation science.	Progress will depend on scalable personalized prevention, integration of mental health and reporting culture into care pathways, and follow-up across the transition to adulthood.

## 2 Physiological basis of injury: from growth plate vulnerability to tissue adaptability

The adolescent musculoskeletal system is characterized by a dynamic process of growth and maturation. Composed of chondrocytes, growth plates serve as active sites for longitudinal bone growth and possess substantially lower mechanical strength than adjacent bone tissue. Repetitive stress renders growth plates vulnerable structural weak points, predisposing them to epiphyseal injuries or apophysitis (Armento et al., 2023). For example, repetitive torsional and tensile stresses on proximal humeral growth plates during throwing activities may cause ‘Little League shoulder’, fundamentally a Salter-Harris type I fracture of the proximal humeral epiphysis (Lintner et al., 2023). Similarly, the tibial tuberosity (Osgood-Schlatter disease) and patellar tendon insertion (Sinding-Larsen-Johansson disease) in the knee joint, as traction apophyses, are prone to inflammation and micro-tears under powerful, repetitive quadriceps contractions, forming clinically common anterior knee pain syndrome (Wada et al., 2024; Wedderkopp et al., 2026).

Beyond structural vulnerability, the hormonal milieu during adolescence exerts a decisive influence on skeletal health and tissue adaptability. Estrogen and testosterone play pivotal roles in promoting bone mineralization and enhancing bone mineral density. Functional hypothalamic amenorrhea is not uncommon among female adolescent athletes. Its core mechanism involves suppression of the HPO axis function due to insufficient energy availability, leading to a low-estrogen state (Wong et al., 2025). This state directly impairs the critical bone mass accumulation process during adolescence, resulting in reduced bone mineral density and compromised bone microarchitecture, thereby significantly increasing the risk of stress fractures (Armento et al., 2023; Atadja et al., 2024; Wong et al., 2025). Recent studies have further identified a similar pathophysiological triad in male athletes, characterized by low energy availability, hypogonadism, and reduced bone mass, highlighting the importance of energy balance for skeletal health in adolescent athletes of both sexes (Armento et al., 2023).

Adaptive responses and imbalances in tissues constitute another key physiological mechanism underlying overuse injuries. Training aims to induce positive adaptive changes in muscles and tendons, such as increased muscle strength and enhanced tendon stiffness. However, these adaptations may occur asynchronously. Studies have identified a potential ‘imbalance’ between muscle strength and tendon stiffness in adolescent athletes (Domroes et al., 2024; Domroes et al., 2025). When muscle strength develops rapidly while tendon stiffness lags relatively, the strain (deformation) experienced by tendons during maximal contractions increases. This may exceed the tendon’s safe load-bearing range, elevating the risk of tendinopathy or rupture. A study on adolescent female handball players successfully reduced the incidence of muscle-tendon imbalance in knee extensor muscles through personalized load intervention, demonstrating that targeted training can elicit more coordinated tissue adaptation (Domroes et al., 2025). At the molecular level, recent animal model studies have revealed potential mechanisms for sex differences: under equivalent fatigue loads, female mice exhibit greater collagen matrix damage in the anterior cruciate ligament (ACL) than males, with slower and more finely regulated repair responses. In contrast, males demonstrate enhanced proliferative and migratory cellular responses (Schlecht et al., 2026). These potential physiological differences in injury response may partially explain the higher incidence of ACL injuries and re-injury rates among female athletes.

## 3 Multidimensionality of risk factors: interplay of early specialization, skeletal maturity, gender differences, and training load

The occurrence of sports injuries in adolescents results from complex interactions among multiple endogenous and exogenous risk factors. Understanding this multidimensionality is a prerequisite for developing effective prevention strategies.

Early sport specialization has become an increasingly recognized independent risk factor. It is typically defined as year-round focus on a single sport with exclusion of other sports, often accompanied by high-intensity training. Substantial evidence indicates that early specialization is significantly associated with increased risk of overuse injuries (Li et al., 2023; Nagano et al., 2023; Buser et al., 2025; Thurber et al., 2026). The mechanism may involve repetitive singular movement patterns causing sustained stress on specific tissues without adequate recovery or varied stimuli, while simultaneously limiting the comprehensive development of overall motor skills and physical fitness. Early Specialization is particularly prominent in individual sports such as tennis and gymnastics (Thurber et al., 2026). A retrospective study of Japanese athletes revealed that in team sports (e.g., soccer, basketball), athletes who maintained specialization from elementary through high school demonstrated a significantly higher prevalence of Overuse injury during high school compared to non-specialized athletes (Nagano et al., 2023).

Biological age or skeletal maturity constitutes another core intrinsic factor. Skeletal age frequently demonstrates asynchrony with chronological age, placing adolescents in accelerated growth phases at elevated risk. Evaluation of skeletal maturity through methodologies like predicted adult height percentage facilitates enhanced precision in injury risk assessment (Davis et al., 2023). Studies observe distinct injury patterns across maturation stages: growth-related injuries (e.g., apophysitis) peak during early-to-mid adolescence, whereas muscle strains and joint sprains demonstrate higher prevalence in advanced maturity stages (Monaco et al., 2026). In male adolescent soccer players, the overall incidence, severity, and burden of injuries increase with age, with skeletal injuries peaking during the U14 age group, corresponding to the peak adolescent growth spurt period (Weishorn et al., 2023).

Marked gender differences exist in injury epidemiology, stemming from physiological variations across multiple dimensions including anatomical, hormonal, and neuromuscular control factors. Systematic reviews confirm that female athletes face elevated risk compared with males in the same sports for knee injuries, foot/ankle injuries, bone stress injuries, and concussion; Conversely, male athletes face higher risks of hip/groin injuries, hamstring injuries, and acute upper limb fractures (Hardaker et al., 2024). In high-contact sports such as rugby, adolescent females demonstrate significantly higher overall injury rates, concussion rates, and injury rates during tackling compared to males (Shill et al., 2023; West et al., 2023a). This discrepancy extends beyond incidence; the previously discussed differences in molecular-level repair responses suggest post-injury physiological recovery processes may also exhibit sex-specific characteristics (Schlecht et al., 2026).

Training load (including volume, intensity, and frequency) constitutes the primary modifiable extrinsic factor. Training volume exceeding tissue repair and adaptive capacity is the direct trigger for overuse injury. Training hours per week exceeding biological age is commonly used as a simple indicator for high-risk loading (Li et al., 2023). Sudden increases in training and competition loads, insufficient recovery, and incompletely rehabilitated preexisting injuries during the preseason period have all been identified as significant risk factors for new injuries during the competitive season (Sonesson et al., 2023). Notably, early specialization, high training loads, and specific maturation stages often converge to collectively amplify injury risk. For instance, young male basketball athletes exhibiting taller stature and high specialization demonstrate a significantly elevated risk of reporting growth-related knee pain (Koyama et al., 2026).

## 4 Evolution of injury prevention and management strategies: from neuromuscular training to personalized programs

Driven by enhanced understanding of injury risk factors, prevention strategies have evolved from generalized approaches to context-driven, personalized interventions.

Neuromuscular training currently represents one of the best-evidenced and most widely implemented injury prevention strategies. Such protocols typically integrate strength, balance, agility, and sport-specific technical training aimed at optimizing movement patterns, enhancing joint stability, and improving tissue load tolerance. In team sports such as soccer (e.g., the ‘FUNBALL’ program) and Rugby, neuromuscular training has demonstrated efficacy in reducing overall injury incidence, particularly severe injuries (West et al., 2023b; Obërtinca et al., 2025). For adolescent female athletes with a history of concussion, while neuromuscular training is effective, its relative efficacy in reducing the risk of lower-extremity musculoskeletal injuries may be less pronounced compared to athletes without concussion history, suggesting that high-risk subgroups may require enhanced or differentiated intervention measures (McPherson et al., 2024).

Personalized prevention and management represent the current cutting-edge approach. The core concept involves identifying individual-specific risk factors (e.g., muscle-tendon imbalance, restricted joint range of motion, biomechanical deficiencies) and designing targeted interventions accordingly. For instance, by assessing patellar tendon strain during maximal voluntary contraction of the knee extensors, athletes exhibiting distinct imbalance patterns such as ‘insufficient tendon stiffness’ or ‘inadequate muscle strength’ can be identified. These individuals can then be assigned personalized loading training programs specifically designed to prioritize either tendon adaptation or muscle adaptation (Domroes et al., 2024; Domroes et al., 2025). This precision-based approach has demonstrated favorable outcomes in reducing the incidence of Muscle-Tendon Imbalance among adolescent handball players. Similarly, for Fast Bowlers in cricket, management strategies for Lumbar bone stress injury are evolving from generalized ‘functional’ management (primarily symptom-based) toward population-specific ‘structural’ management. This involves monitoring bony union progression through serial MRI to guide rehabilitation, with the aim of achieving bony union rather than fibrous union to attain superior long-term prognosis (Orchard et al., 2023).

The value of education as a crucial component of intervention has become increasingly prominent. Enhancing awareness among athletes, coaches, and parents regarding signs of overuse injuries, growth patterns, and principles of scientific training establishes the foundation for facilitating behavioral changes. A randomized controlled trial involving young volleyball players demonstrated that educational programs significantly improved athletes’ knowledge levels concerning overuse injuries and adolescent growth processes (Lau et al., 2025a). Furthermore, adolescents’ willingness to report injuries is influenced by multiple factors including knowledge, attitudes, and social norms. Strengthening relevant education may mitigate injury concealment and promote early medical attention (Cheever et al., 2024).

## 5 Academic debates and key controversies

Despite ongoing research, significant controversies persist within the academic community regarding multiple key aspects of sports injuries in adolescents.

The primary controversy centers on the optimal treatment strategy for specific injuries, particularly the choice between surgical and non-surgical interventions. For mid-shaft clavicle fractures in adolescents, the vast majority of studies indicate that non-surgical management yields favorable outcomes; however, the boundaries defining surgical indications (such as significant displacement and shortening) remain under discussion (Patel et al., 2023). For adolescents experiencing first-time patellar dislocation, while nonsurgical management remains the preferred approach, ongoing debate concerns whether a high-risk subgroup (e.g., individuals with substantial osteochondral fractures or marked anatomical anomalies) exists that may benefit from early surgical intervention (Sinikumpu et al., 2023). In managing lumbar spondylolysis, divergent views also persist concerning the necessity and precise efficacy of bracing (Helenius et al., 2025; Virkki et al., 2025).

Second, considerable controversy surrounds the trade-offs of early sport specialization. On one hand, specialization is viewed as a potential pathway to elite performance; On the other hand, substantial evidence links it to increased injury risk, athletic burnout, and shortened sports careers (Buser et al., 2025; Thurber et al., 2026). The controversy centers on whether all sports and athletes at all levels should avoid Early Specialization, and how the specific age boundary for ‘early’ should be defined. Some perspectives suggest that a degree of Early Specialization may be necessary in sports with critical early technical windows (e.g., gymnastics, figure skating), but it must be combined with scientific load management and comprehensive physical fitness training.

Furthermore, discussions regarding whether prevention strategies should be ‘generalized’ or ‘highly specific’ are ongoing. Traditional neuromuscular training programs predominantly employ universal designs. While effective, they face compliance issues and a ‘From Research to Practice’ translation gap (Lau et al., 2025b). Consequently, scholars advocate for developing ‘context-driven’ prevention programs that fully consider the unique needs and implementation settings of specific sports, genders, age groups, cultural backgrounds, and even individual teams when designing intervention measures (Shill et al., 2023; Lau et al., 2025b). For example, given the characteristic of higher injury rates among female adolescent rugby players, there are increasing calls to develop female-specific injury prevention strategies (Shill et al., 2023). However, the high development costs and limited scalability of highly specific protocols raise questions about whether their efficacy gains justify the investment, necessitating further validation studies.

## 6 Identifying research gaps

While current research has made significant progress, it has also revealed critical evidence gaps and unresolved questions.

First, research on psychosocial factors and injury reporting behavior remains in its infancy, lacking effective intervention strategies. Injury experiences among adolescent athletes are frequently accompanied by psychological issues such as anxiety and depression, while a strong athletic identity may exacerbate post-injury psychological distress (Park et al., 2023). However, research on screening tools and effective intervention measures for the psychological health of adolescent athletes post-injury is markedly scarce. Simultaneously, adolescents often conceal injuries due to fear of losing playing opportunities or disappointing their team or coaches (Cheever et al., 2024). Although knowledge, attitudes, and social norms are known to influence reporting willingness, designing effective interventions to modify this behavior and facilitate early diagnosis and treatment remains a significant gap.

Secondly, the underlying biological mechanisms responsible for gender differences remain largely unelucidated. Although epidemiological data clearly demonstrate gender differences in injury patterns, the molecular, cellular, and physiological basis for these differences remains inadequately understood. For instance, while biomechanical factors contributing to females’ higher ACL injury risk have been extensively studied, research on more fundamental mechanisms, such as the gender differences in extracellular matrix damage and repair responses revealed by the aforementioned animal experiments (Schlecht et al., 2026), remains limited. Longitudinal studies are also needed to clarify how hormones (e.g., Estrogen, relaxin) dynamically influence the material properties and injury responses of tendons and ligaments.

Third, long-term outcome data are severely lacking. The vast majority of studies focus on short-term management of injuries and return-to-play timelines; however, we know very little about whether and how sports injuries during adolescence affect adult joint health, functional capacity, and quality of life. For example, what is the risk that incompletely healed adolescent lumbar spondylolysis or knee osteochondral injuries will lead to degenerative joint pathologies in middle age? Does ‘structural’ management of lumbar bone stress injuries in cricket fast bowlers genuinely extend their high-level athletic careers (Orchard et al., 2023)? Addressing these questions requires decades-long prospective cohort studies, for which current evidence remains virtually nonexistent.

Finally, the lack of standardized diagnosis and classification hinders research comparability. This is particularly prominent in some relatively rare injuries. For instance, among adolescent rock climbers with phalangeal apophyseal stress injuries, the absence of a widely accepted imaging classification system presents considerable challenges for cross-study comparisons and meta-analyses (Schöffl et al., 2025). Uniform and operational diagnostic and severity grading criteria form the foundation for advancing research in any injury domain.

## 7 Conclusions and future directions

Musculoskeletal injuries related to youth sports activities constitute a multifactorial issue driven by complex physiological mechanisms. Current research has progressed from characterizing epidemiological patterns to investigating fundamental domains, including growth plate biology, tissue adaptation and maladaptation, hormonal regulation, and sex-specific physiological responses. Contemporary risk factor models incorporate increasingly sophisticated frameworks that account for multifaceted interactions among early specialization, skeletal maturity, sex differences, and training load variables. Prevention paradigms are shifting toward personalized, context-driven methodologies, with neuromuscular training, athlete education, and individualized load management constituting the foundational elements of integrated intervention strategies.

Nevertheless, ongoing academic debates underscore the complexity inherent in clinical decision-making, while significant research lacunae delineate critical future investigative priorities. Looking ahead, research and practice in this field will advance along the following high-potential directions:

First, deep interdisciplinary integration. Future research must transcend disciplinary boundaries between sports medicine, developmental biology, biomechanics, endocrinology, psychology, and rehabilitation engineering. For instance, multidimensional individual injury risk prediction models should be developed by integrating exercise load monitoring, hormone level detection, genetic polymorphism analysis, and advanced radiomics. A comprehensive understanding and management of the Triad (female and male) in athletes necessitates close collaboration among sports medicine physicians, nutritionists, endocrinologists, and psychologists (Armento et al., 2023; Wong et al., 2025).

Second, advancing precision and personalized medicine. The development of personalized injury risk warning and intervention strategies based on biomarkers, wearable device data, and AI algorithms will be at the forefront. Research should focus on developing cost-effective, personalized assessment tools [e.g., improving the risk assessment tool in Reference (Schley et al., 2025)] and training protocols that are easy to implement at the community level.

Third, filling research gaps in long-term outcomes and translational research. There is an urgent need to initiate large-scale, long-term prospective cohort studies tracking adolescent athletes into adulthood or even middle age to clarify the long-term sequelae of early sports injuries. Concurrently, research in implementation science should be strengthened, focusing on exploring how to integrate evidence-based preventive measures (such as Neuromuscular Training) into adolescent sports training systems across different levels and contexts with high fidelity and high compliance, thereby truly bridging the gap from Research to Practice (Lau et al., 2025b).

Fourth, strengthening attention to psychosocial dimensions. Routinely integrating mental health screening and interventions into the post-injury management process for adolescent athletes, and developing effective strategies to encourage a safe sports participation culture and timely Injury Reporting Behavior, are crucial for safeguarding their physical and mental well-being (Park et al., 2023; Cheever et al., 2024).

In conclusion, protecting adolescent athletes from sports injuries to ensure their safe and sustainable benefit from athletic activities constitutes a systematic undertaking that demands ongoing scientific innovation, multidisciplinary collaboration, and collective participation across societal sectors. By elucidating physiological mechanisms and strategically addressing extant knowledge gaps, we advance toward an epoch characterized by precision prevention and optimized management of sports injuries among adolescents.
